# The role of professional socialisation in confidence in vaccines and vaccination decision-makers: Insights from a large multi-wave survey in France

**DOI:** 10.1371/journal.pone.0328548

**Published:** 2025-08-12

**Authors:** Hugo Touzet, Sophie Privault, Jeremy K. Ward

**Affiliations:** CERMES3 (INSERM, CNRS, EHESS, Université de Paris), Villejuif, France; National Institute of Demographic Studies: INED, FRANCE

## Abstract

The Covid-19 pandemic has greatly expanded research on social determinants of health inequalities. Yet one crucial dimension remains underexplored: the influence of professional socialisation (i.e., the process through which individuals acquire not only knowledge and skills, but also a worldview and a culture associated with the profession they practice). This gap is striking given the extensive sociological evidence that professional identities profoundly shape individuals’ life paths, perceptions, and health experiences. In this article, we take advantage of a very large multi-wave survey conducted in France during the Covid-19 pandemic (n > 100,000) to explore in greater depth the relationship between occupation and attitudes to vaccination and to stakeholders involved in vaccination policymaking. We show that, controlling for various socio-demographic factors, major disparities emerge, not only between broad professional groups at different places in the social hierarchy, but also between professions with comparable situations in this hierarchy. For instance, we show that public sector employees are more in favour of vaccinations but less confident in the government than their private sector counterparts. To understand these differences, we draw on the sociology of the relationship between professional socialisations and ordinary relationships to politics and the State.

## Introduction

The Covid-19 pandemic affected virtually every country, every economic sector and every population. Very quickly, work in the social sciences highlighted marked social inequalities in the experiences of this crisis. For instance, major disparities were revealed in vaccination coverage, experience of lockdowns, impact on mental health and on economic and social conditions [1,2].

Vaccination has become an object of focus in the literature on inequities in health with studies showing that coverage, attitudes and experiences are influenced by race, income, qualifications, place of residence, political marginalisation, etc. [3–6]. These factors bear on people’s social trajectory, and socialisation which determine their perception of the epidemic threat, their access to vaccines, the trust they have in official recommendations, in health authorities more generally and/or the type of discourse they have access to. On this front, the Covid-19 epidemic represented a leap in the study of the social determinants of a wide range of dimensions of health, including adherence to official recommendations.

Yet one crucial dimension remains underexplored: the influence of professional socialisation. This gap is striking given the extensive sociological evidence that professional identities profoundly shape individuals’ life paths, perceptions, and health experience. Professional socialisation is the process through which individuals acquire not only knowledge and skills, but also a worldview and a culture associated with the profession they practice. One’s profession or occupation can strongly influence their experience of vaccination as it can determine their exposure to infectious risk, the material resources available to deal with such risks, but also the consequences for them of restrictive measures such as lockdowns or green passes. The Covid-19 crisis was characterised by a multitude of professional experiences, ranging from “key workers” on the frontline of the fight against the epidemic [7–9], adapting to teleworking [10], to going through extended periods of inactivity.

The exception to this relative dearth of research into the links between vaccination and professional identities and experiences is the case of healthcare workers. Studies have shown that, depending on their occupation, attitudes to vaccines differ among healthcare workers. This is particularly marked in France, with a strong “occupational gradient” of reticence [11], but this phenomenon can also be observed in other countries, such as Israel [12,13]. In France, differences were also observed depending on the type of practice (self-employed or employee), in the context of a crisis in the funding of the public healthcare system [14]. In France and Israel, working conditions, job satisfaction and hierarchical relationships have had an impact on the selection of sources of information, trust in health authorities and in the workplace hierarchy during the Covid-19 crisis [12,15]. Studies of vaccine hesitancy among healthcare workers therefore highlight the fact that professional socialisation (i.e., the implicit and explicit learning that makes it possible to enter and master an occupation) and the experience of workplace relations affect attitudes to health beyond the strict question of epidemic exposure. They are in line with classic analyses in the sociology of professions and occupational groups, which have clearly documented that professional socialisation is an essential aspect of the construction of individual trajectories and experiences, including with regards to health [16]. In particular, research has shown that they have an influence that goes beyond issues specific to the work people do [17]. Recent work has also highlighted the influence of the “organisational climate” – made up of the shared perceptions that individuals have of their work environment – on employees’ compliance with anti-Covid-19 measures [18] and acceptance of vaccines against Covid-19 [19].

The aim of this article is to take advantage of a very large multi-wave survey conducted in France during the Covid-19 pandemic (n > 100,000) to explore in greater depth the relationship between occupation and attitudes to vaccination and to actors involved in vaccination policymaking. This database follows the French statistical nomenclature, which breaks down occupations into 18 categories. Using a much less detailed approach, recent studies have already begun to highlight the role of occupations in inequalities in health and vaccination in France during the Covid-19 epidemic [20–23]. For instance, vaccine coverage against Covid-19 was 11 points higher among *managers and people in higher intellectual occupations* than among *manual workers* in the fall of 2022 [24]. It was also shown that perceptions of epidemic risk and other aspects of the epidemic were influenced by people’s professional background [25,26].

In this article, we extend these initial studies by adopting a more fine-grained approach of the relationship between occupations and health. We focus on attitudes to vaccination and confidence in two types of stakeholders directly linked to public health management: the government and scientists. More specifically, we show that major inequalities emerge, not only between broad professional groups at different places in the social hierarchy, but also between professions with comparable situations in this hierarchy. By social hierarchy, we refer to the structured ranking of social positions based on factors such as occupational status, income, education, and symbolic recognition. It reflects both material inequalities and perceived social value within a given society. We show that belonging to an occupation associated with the upper classes is associated with greater acceptance of vaccination, and greater confidence in the ability of the government and scientists to tackle the epidemic. But more importantly, we show that public sector employees are more in favour of vaccinations but less confident in the government than their private sector counterparts. To understand these differences, we draw on the sociology of the relationship between professional socialisations (the process through which individuals acquire not only knowledge and skills, but also a worldview and a culture associated with the profession they practice) and ordinary relationships to politics and the State.

## Data and method

### Ethical approval statement

The EpiCov study was approved by the CNIL (the French independent administrative authority responsible for data protection, ref.: MLD/MFI/AR205138) and by the Comité de protection des personnes (the French equivalent of the Research Ethics Committee). The EpiCov study has also been approved by the Official Statistics Label Committee, attesting to its compliance with statistical quality standards. Participants’ informed consent was duly obtained via mail, e-mail or SMS. For more information, see the website of the EpiCov study: https://drees.solidarites-sante.gouv.fr/sources-outils-et-enquetes/enquete-epicov-epidemiologie-et-conditions-de-vie-sous-le-covid-19.

### Study design

The EpiCov survey (“Epidemiology and Living Conditions”) is a multi-wave cohort survey set up in April 2020 with the aim of understanding the main epidemiological, social and behavioural issues linked to the Covid-19 pandemic in France. The survey was approved by the CNIL (the French independent administrative authority responsible for data protection, ref.: MLD/MFI/AR205138) and by the Comité de protection des personnes (the French equivalent of the Research Ethics Committee). The survey has also been approved by the Official Statistics Label Committee, attesting to its compliance with statistical quality standards. The cohort protocol is detailed in another publication [27]. A random sample of 135,000 people aged 15 and over, drawn from the tax database (Fidéli) of the Institut national de la statistique et des études économiques (INSEE), which covers 96% of the population living in France, took part in a first wave of the study from 02/05/2020 to 01/06/2020 (T1). A second wave was carried out from 26/10/2020 to 08/12/2020 (T2), in which 107,808 respondents took part (81.7% of respondents from wave 1). A third wave was conducted in from 24/06/2021 to 07/08/2021 (Q3), in which 85,032 respondents took part (78.9% of respondents from wave 2). The same individuals were contacted for each wave of the survey; however, a natural decline in participation occurred over time. Individuals were invited to complete the questionnaire online, or by telephone for those who did not have internet access.

### Dependent variables

We focus on respondents aged 18 and over and on waves 2 and 3, which contained the following questions on vaccination and actors involved in vaccination policymaking:

- Do you intend to be vaccinated against Covid-19? « Yes, certainly », « Yes, maybe », « Probably not », « Certainly not ». (Answers were recoded as Yes or No with « I Don’t know » included among the latter);
- Are you very, rather, rather not or not at all in favour of vaccinations in general? (recoded as « favourable » or « not in Favour »);
- To limit the spread of the coronavirus, do you have confidence in the action of the government? « Yes, certainly », « Yes, rather », « No, rather not », « No, not at all » (recoded as « yes » or « no »);
- To limit the spread of the coronavirus, do you have confidence in the ability of scientists? « Yes, certainly », « Yes, rather », « No, rather not », « No, not at all » (same as above);

Wave 1 did not include items on vaccination and was therefore excluded from analysis which centers on waves 2 and 3. For clarity, we focus on wave 2 in the main text and present the analyses of wave 3 in the supplementary materials as they are very similar to results for wave 2.

### Context of waves 2 and 3 of the EpiCov study

Waves 2 and 3 of the EpiCov study were conducted at two very different moments in the Covid-19 crisis and the unfolding of the Covid-19 vaccination campaign (see Anonymous 2 for a detailed description). While concern over Covid-19 and support for strong measures against the epidemic were very high during most of the first lockdown in the spring of 2020, both dropped considerably during the rest of the year [22,23,28]. This rise in complacency, pandemic fatigue and distrust in the French government’s handling of the epidemic was reflected in the evolution of intentions to vaccinate against Covid-19 when a vaccine would be available. The share of French adults who intended to vaccinate dropped from around 80% in March 2020 to around 45% in December 2020 when the vaccination campaign was officially launched [22] (Anonymous 2). The second wave of EpiCov conducted in October to early December of that year therefore captures the moment when support for Covid-19 vaccination was the lowest and doubts were the highest even though this was also the moment when information on the vaccines and the vaccination campaign started surfacing. In the months following the launch of the vaccination campaign, the share of those willing to be vaccinated and of those who already had a first dose grew steadily, reaching 80% in July 2021 when a Pass Sanitaire was announced. This pass allowed people who were vaccinated or who could present a negative Covid-19 test to access a great number of public places including bars, restaurants, nursing homes and hospitals. This pass enabled France to reach a high vaccine coverage of around 93% of adults by the autumn [29,30]. However, many of the vaccinated felt constrained into doing so and remained very skeptical of this vaccination [31–33]. Consequently, the third wave of EpiCov captures another crucial moment. It was conducted around the time when this health pass was announced on the 12^th^ of July but most respondents answered before the President’s speech which really signalled that constraints would be put in place (around 77% of our sample, and around 79% of healthcare workers).

### Variable of interest: The « Occupations and socio-professional categories » nomenclature (PCS)

The importance of occupational nomenclatures is widely recognised in the analysis of social phenomena. Several are widely used across the world, such as the ISCO nomenclature (International Standard Classification of Occupations) managed by the International Labour Organization (ILO), the ESeG nomenclature (European Socio-economic Groups), from the European statistics institute Eurostat, or the EGP scheme (Erikson-Goldthorpe-Portocarero), from which the UK classification system, the National Statistics-Socio Economics Classification (NS-SEC), is adapted. In France, the PCS (Occupations and socio-professional categories) is the statistical nomenclature of reference for analysing professional activities in relationship to social stratification. It was designed by the National Institute of Statistics and Economic Studies (INSEE). In principle, it differs from ISCO in that it takes into account not only the nature of work practices, but also the economic and institutional environment, thus defining social positions linked to occupational situations. In particular, it makes it possible to distinguish workers in the public sector from those in the private sector, or the self-employed from employees. Its construction encompasses a range of parameters (employment status, sector of activity, type of employer, level of qualification, etc.) [34,35]. This makes it a particularly interesting indicator for representing the social space and related inequalities. Belonging to a ‘category’ is in reality a set of experiences of work, hierarchy and insecurity that influence attitudes and behaviours. It is for this reason that it is an important predictor of a wide range of attitudes, including towards politics [36].

The developed version of the PCS comprises 18 categories provided in the EpiCov survey (« PCS categories *»* in the rest of the article, see Table 1. for the list) but researchers usually focus on the aggregated version of the nomenclature which reduces it to six « socio-professional groups » (« PCS groups » in the rest of the article). However, to understand the effect of occupations precisely, it is necessary to use the former, more detailed, version of the nomenclature. For instance, sociologists have shown that within the broad family of *Employees* or *Manual workers* there are in fact different “professional spheres” [37]. Similarly, political behaviour among *Managers and higher intellectual occupations* and *intermediate professions* varies, for example according to whether they work in the private or public sector [38]. More generally, many researchers have highlighted the value of an approach based on occupations or micro-classes rather than ‘big classes’ [39,40].

**Table 1:** Association between PCS and attitudes to vaccines, the government and scientists during Covid-19 – multivariate logistic regression (November 2020).

		Are you very, rather, rather not or not at all in favour of vaccinations in general? Wave 2, N = 91211		Do you intend to be vaccinated against Covid-19? Wave 2, N = 90611		To limit the spread of the coronavirus, do you have confidence in the action of the government? Wave 2, N = 90012		To limit the spread of the coronavirus, do you have confidence in the ability of scientists? Wave 2, N = 90073	
		Total in favour	OR	Total yes	OR	Total yes	OR	Total yes	OR
**Gender**									
Women		70%	0,75***	54,5%	0,5***	49%	0,94***	76%	0,84***
Men		75%	**REF**	70%	**REF**	52%	**REF**	80%	**REF**
**Age**									
18-24		75%	0,92*	59%	0,95	42%	0,71***	78%	0,92*
25-34		68%	0,82***	49%	0,75***	39%	0,73***	73%	0,86***
35-49		70,5%	**REF**	55%	**REF**	47%	**REF**	76%	**REF**
50-64		72%	1,11***	64%	1,46***	53%	1,38***	79%	1,4***
65-74		78%	1,3***	75%	2,01***	60%	1,73***	82%	1,57***
75 and more		81%	1,77***	78%	2,5***	64%	2,11***	81%	1,63***
**Income**									
min-d1		66,5%	0,66***	57%	0,73***	45%	0,66***	73%	0,72***
d1-d2		64%	0,63***	55%	0,7***	41%	0,61***	70%	0,68***
d2-d3		66%	0,65***	54%	0,66***	43,5%	0,65***	73%	0,75***
d3-d4		68,5%	0,69***	56%	0,69***	44%	0,66***	73%	0,75***
d4-me		70%	0,74***	59%	0,71***	47%	0,69***	74,5%	0,76***
me-d6		69%	0,73***	58%	0,71***	47%	0,69***	75%	0,77***
d6-d7		70,5%	0,77***	59%	0,75***	47%	0,72***	76%	0,86***
d7-d8		72%	0,78***	59%	0,74***	49%	0,77***	78%	0,89***
d8-d9		75%	0,84***	63,5%	0,81***	53%	0,84***	81%	0,97***
d9-max		80%	**REF**	71%	**REF**	60%	**REF**	84%	**REF**
**Level of education**									
Less than a High school diploma		66,5%	**REF**	58%	**REF**	48%	**REF**	73%	**REF**
High school diploma		70%	1,21***	57%	1,22***	47%	1,11***	77%	1,3***
Bachelor degree		75%	1,58***	62%	1,53***	51%	1,25***	80%	1,61***
Master degree and doctoral students in healthcare		84%	2,44***	73%	2,31***	58%	1,49***	86%	2,17***
Doctoral students outside healthcare		90%	3,89***	85%	3,69***	60%	1,43***	89%	2,71***
**PCS**									
**Farmers**	*10. Farmers*	66%	0,81*	56%	0,92	50%	1,26***	74%	0,91
**Self-employed and entrepreneurs**	*21. Craft workers*	60%	0,66***	48%	0,74***	42%	0,97	71%	0,85*
	*22. Shopkeepers*	63%	0,75***	52%	0,94	44%	1,03	73%	0,93
	*23. Buisness owners g*	77%	0,88	71%	1,06	60%	1,36***	78%	0,77*
**Senior executives and higher professionals**	*31. Liberal profession*	77%	0,99	66%	1,2***	51%	1,08	81%	1,03
	*32. Public service executives and intellectual and artistic professions*	83%	1,33***	71%	1,45***	53%	1,16***	86%	1,35***
	*36. Business executives*	81%	1,11*	70%	1,28***	61%	1,62***	85%	1,29***
**Intermediate Professions**	*41. Intermediate professions in education, health and the public service or similar*	74%	1,13**	55%	1,11**	44%	0,99	77%	1,02
	*46. Corporate intermediate administrative and commercial professions*	73,5%	1,03	59%	1,18**	51%	1,32***	79%	1,06
	*47. Technicians*	68%	0,8***	56%	0,94	44%	1,07	76%	0,97
	*48. Forepersons*	67%	0,8***	57%	1	50%	1,27***	77,0%	1
**Employees**	*51. Public sector clerical and service workers*	68,5%	**REF**	50%	**REF**	42,5%	**REF**	74,5%	**REF**
	*54. Corporate administrative workers*	64%	0,78***	45%	0,86***	47%	1,2***	73%	0,90*
	*55. Retail workers*	61,5%	0,8***	46%	0,96	40%	1,05	70%	0,90*
	*56. Service workers pg*	64%	1,03	45%	1,08	42,0%	1,07	70%	0,96
**Manual workers**	*61. Skilled manual workers*	61%	0,75***	51%	0,9**	39,5%	0,97	68,5%	0,83***
	*66. Unskilled manual workers*	57%	0,73***	44%	0,86**	36%	0,91	65%	0,79***
	*69. Agricultural workers*	65%	0,92	52%	1,01	43%	1,17	70%	0,92
**Students**		80%	1,9***	64%	1,84***	46%	1,59***	83%	1,83***
**Unemployed**		64%	0,85***	52%	1,07	40%	1	70%	0,85***
**Retired**		78%	1,24***	74%	1,59***	59%	1,24***	81%	1,1*

**Guide to reading the table:** 57% of unskilled manual workers are in favor of vaccinations in general. They are 27% less likely to be in favor of vaccinations than public sector clerical and service workers, with an odds ratio of 0.73 (p < 0.001) (model controlled by age, gender, income and level of education, see methodology section).

Several studies have used the EpiCov survey to explore the influence of the PCS, but they have done so at the aggregated level of socio-professional *groups*. In this article, we extend these analyses by using the PCS variable at a finer level, that of socio-professional *categories*, with 18 modalities.

### Statistical analyses and control variables

The statistical analysis consists of two parts.

Firstly, we performed cross-tabulations on each outcome by crossing them with a set of other socio-demographic variables (i.e., gender, age, level of education, income, educational attainment, see Table A1 in S1 File for the modalities of these variables) as well as the PCS categories variable which combines the 18 socio-professional *categories*, and *students*, *the unemployed* and *the retired* (21 modalities).

We chose to exclude respondents who did not answer all the questions associated with these variables. But, in order to retain the largest possible number of respondents per outcome, we chose to use one sample per outcome. The size of the sample therefore varies depending on the outcome and wave (72324 < n < 91211, see Tables A1 and A2 in S1 File for the samples’ compositions, and Tables B1 and B2 in S2 File for cross-tabulation with all outcomes). Compared to official data sensus from the INSEE, the sample composition does not reflect exactly that of the French adult population: the least educated respondents, those in management and higher intellectual occupations and the richest are over-represented while respondents aged 75 and more and the unemployed are under-represented. Nevertheless, these differences are limited and all groups present a great number of respondents.

Secondly, we performed binomial logit regressions to test the significance of the differences observed between the PCS categories in the cross-tabulations controlling for gender, age, level of education and income (See Table 1 and Tables C1–C4 in S4 File for results for wave 2 and Tables D1–D4 in S5 File for wave 3). In order to test the significance of differences between close PCS categories (within each aggregated PCS group), each version of the regression was run four times, each time with a different reference value*: Executives in the public service, Intellectual and artistic professions*; *Intermediate professions in education, health, the public service and similar occupations*; *Public sector clerical and service workers*; *Skilled manual workers* (see Tables C1–C4 in S4 File and D1–D4 in S5 File). We also performed ordinal logistic regressions (Tables E1–E4 in S6 File and F1–F4 in S7 File). In the rest of the text, we will focus on the results of the binomial logistic regressions and evoke the ordinal regressions when their results are different or complementary.

Finally, the size of the samples allowed us to pay a particular attention to healthcare workers. This part of the analysis will only consist of descriptive statistics. In the survey, healthcare workers refer to both nurses and doctors. To separate these two professions, we divided this group according to educational attainment (holding a master degree and/or a doctorate in health vs having a bachelor degree or high school diploma) (see Table 2).

**Table 2:** Focus on key occupations.

	Are you very, rather, rather not or not at all in favour of vaccinations in general?, Wave 3, N = 6455		Vaccination, Wave 3, N = 6455		To limit the spread of the coronavirus, do you have confidence in the action of the government?, Wave 3, N = 6451		To limit the spread of the coronavirus, do you have confidence in the ability of scientists?, Wave 3, N = 6451	
	Total In Favour	Total N	Total yes	Total N	Total yes	Total N	Total yes	Total N
Nursing assistant, paramedical staff	83%	1566	84%	1566	52%	1565	87%	1565
Proxy Nurse (bachelor degree or high school diploma)	90%	1254	88%	1254	56%	1254	92%	1254
Proxy Doctor (master degree and/or a doctorate in health)	95%	605	95%	605	69%	604	97%	604
Firefighter, first-aider, ambulance driver	83%	161	81%	161	50%	161	83%	161
Pharmacist	93%	277	91%	277	64%	277	93%	277
Police officer	84%	307	84%	307	54%	307	86%	307
Teacher	90%	2285	89%	2285	61%	2283	94%	2283

## Results

Between wave 2 (November 2020) and wave 3 (July 2021), we observe important variations in responses to our four outcomes. While only 61% of our sample in wave 2 intended to vaccinate against Covid-19, 86% of the sample in wave 3 had been vaccinated or intended to do so. The share of respondents who have confidence in the ability of the government to limit the spread of the coronavirus rises from 50% to 60%, while the share who have confidence in scientists rises from 78% to 91%. The share of respondents who are in favour of vaccines also increases from 73% to 86%. These evolutions have been analysed elsewhere [20] and are coherent with findings from other surveys conducted during this period (Anonymous 2). They reflect an overall improvement of judgments on the Covid-19 vaccines and the vaccination campaign which can reflect the fact that knowledge of these vaccines progressed as more studies were published and the experience of other countries were widely reported in the news. People had an increasing number of their closed ones who became vaccinated which could appear as both reassuring. The reinforcement of vaccination as a norm, and more waves of Covid-19 unfolded with new variants which may have reinforced the idea that Covid-19 remained a real threat to society (see anonymous 2 for a review of interpretations for this trend) also explained this progression. Despite these evolutions, we found that differences in attitudes according to respondents’ occupation varied very little from one wave to the other, which is consistent with other studies conducted in France showing a relatively stable effect of sociodemographic factors [22,41]. For clarity, we will focus on results from wave 2 (see Tables B2 in S2 File and D1 to D4 in S5 File for models on outcomes from wave 3).

### The socio-professional gradient of vaccination

Our first result regarding the role of occupations is that their social hierarchy is reflected in attitudes to vaccines and confidence in institutions. In all our models (see Table 1. for models on outcomes from wave 2 and Tables B2 in S2 File and D1 to D4 in S5 File for models on outcomes from wave 3), we see a same pattern where respondents in PCS categories associated with lower positions in the social hierarchy (those included in the *Employees* and *Manual workers* groups) tend to have more negative attitudes to vaccines and institutions than respondents in PCS categories associated with intermediate positions (*Intermediate Professions*) which in turn tend to have less positive attitudes than those associated with the highest social positions (*Managers and higher intellectual occupations*).

For example, with regard to opinion on vaccinations in general, 81% *of Managers and those in higher intellectual occupations* said they were in favour of vaccinations in general, compared with 71% of respondents belonging to an *intermediate profession*, 65.5% of *employees* and 60% of *manual workers*. As for the intention to vaccinate against Covid-19 in November 2020, the share of positive responses is 69.5% among *Managers and higher intellectual occupations*. It then drops to 56% among *intermediate professions* and even to below 50% among *employees* and *manual workers*. Similar differences are observed regarding confidence in the government’s action to limit the spread of the virus. Confidence in the scientists’ ability is much more widespread but we also observe significant differences in the share of positive attitudes, ranging from 67.5% for the lowest rate (*manual workers*) to 84.5% for *Managers and higher intellectual occupations*.

### Occupations beyond social hierarchy

More interestingly, we also observe significant differences between occupations that are comparable in terms of social status. These patterns vary depending on our outcomes.

For example, between the PCS categories higher up the social ladder, the *liberal professions* say they are less in favour of vaccines than *public service executives and intellectual and artistic professions* (−6 points/ OR=0.74***, see Table C3 in S4 File). Regarding vaccination against Covid-19, within the *Managers* group, the *liberal professions* are less likely intend to be vaccinated than *public service executives* (−5 points/ OR=0.82***).

A similar observation can be made of the categories occupying a more median position on the occupational scale. *Technicians* and *forepersons* are less favourable to vaccinations in general than *intermediate professions in education, health and the public service or similar* (−6 points and −7 points respectively/ OR=0.71***).

Among *employees*, in terms of intention to vaccinate against Covid-19 there are differences of 4–5 points between those working in the *public sector* (50%) and *corporate administrative workers* (45%, OR=0.86***).

Finally, on the subject of confidence in the government and scientists to limit the spread of Covid-19, differences can be observed, but in a different way than for vaccines. At the top of the social hierarchy, there is a distinction between *public service executives* and *business executives*, the latter being more confident in the government (61% vs 53% OR=1.39***). *Liberal professionals* are less confident in the abilities of scientists than *public service executives* (−5 points/ OR=0.77***).

With regard to the intermediate categories on the PCS scale, there was a significant divide in confidence in the government between *intermediate professions in the public service* (44% confident) and *corporate intermediate administrative and commercial professions* (51% confident, OR=1.33***). Regarding confidence in scientists, the very high levels of confidence are relatively homogeneous between the sub-categories within the intermediate professions, fluctuating between 76% and 79%.

Among *employees*, the level of confidence in the government is also higher in the *corporate administrative workers* category than in the *public sector clerical and service workers* category (47% vs 42.5%/ OR=1.2***). Among both the *employees* and *manual workers*, confidence in scientists varies very little, and the differences are not significant in the model.

These results are confirmed and refined by the ordinal regressions (Tables E1–E4 in S6 File).

For example, compared to those working in the private sector, individuals in the public sector—whether *Employees, Intermediate Professionals*, or *Managers and those in higher intellectual occupations*—are more likely to shift from ‘Rather in favour’ to ‘Very in favour’ regarding vaccines and from ‘Yes, maybe’ to ‘Yes, certainly’ regarding their confidence in scientists. Respondents in the private sector are more likely to shift from ‘Yes, maybe’ to ‘Yes, certainly’ when it comes to the confidence in the government, compared to those in the public sector.

Regarding the intention to get vaccinated, corporate administrative workers are less likely to shift from ‘Don’t know’ to ‘Yes, maybe’ and ‘Yes, certainly’ than their public sector counterparts, contrary to the corporate intermediate administrative and commercial professionals. Additionally, business executives are less likely to shift from ‘Yes, maybe’ to ‘Yes, certainly than public service executives and intellectual and artistic professions.

### A focus on healthcare workers and other « essential » workers

Finally, because of the size of our sample as well as the inclusion of a question aiming to identify essential workers in the EpiCov study, we can present an exploration of the specificities of these occupations. This analysis is restricted to descriptive statistics due to the limited size of each subsample. Respondents were asked whether their occupation was one of the services considered essential and, if so, to specify which one from a list of suggestions. We present the results for the categories “Nursing assistant, paramedical staff”, “Nursing staff, nurse, doctor”, “Pharmacist”, “Firefighter, first-aider, ambulance driver”, “Teacher” and “Police officer” (Table 2). Our comments focus on healthcare professionals. Results for wave 3 are presented in the supplementary materials (Table 2bis in S3 File).

We can see that doctors are much more in favour of vaccinations in general and much more inclined to vaccinate against Covid-19 than nurses, and even more so than paramedics and nursing assistants. Similarly, the former are more confident in the government and scientists than the latter, although the variation is particularly marked for the government (around 17 points differences compared to only 10 for the item of confidence in scientists). Pharmacists are close to doctors on most of these indicators.

## Discussion

Our results confirm and build on pioneering work underlining the importance of occupations and socio-professional situations in people’s attitudes to vaccination and public health decision makers. We have shown that there are major disparities according to occupations. They can be summarised as follows: controlling for age, gender, qualifications and income – which remain important variables – individuals higher up the socio-professional ladder have more positive attitudes to vaccines in general, Covid-19 vaccination, and the government and scientists’ when it comes to their action toward the Covid-19 epidemic. We have also highlighted the fact that social groups are not homogeneous. Within each of the big categories of the nomenclature (*PCS groups*), we see significant variations, particularly between individuals working in the public sector and those in the private sector. While the former are more in favour of vaccinations and more likely to be vaccinated than the latter, they are less confident in the government’s ability to limit the spread of the virus. Finally, in line with these findings, we show that there are also very marked disparities within the healthcare professions.

To begin to explain these disparities, we will firstly focus on the way professional socialisations can directly affect perceptions of health-related issues and then we will focus on how they can indirectly affect them via people’s relationship to the State and politics.

### Occupational differences in the experiences of epidemics and the healthcare system

During the Covid-19 crisis, the whole experience of work was turned upside down. For some, this crisis led to a forced and sudden cessation of work, sometimes for several months at a time, well beyond the strict lockdown periods. While French public policies, through the exceptional deployment of technical unemployment benefits for private sector employees [42], have enabled many of these workers to maintain a decent standard of living, but their daily lives and the way they were organised have been particularly disrupted. On the other hand, precarious workers engaged in undeclared activities (either as their main activity or as a complementary activity) suffered sudden and significant falls in income [43]. For others, often higher up in the social hierarchy, occupying skilled jobs in the tertiary sector for example, the Covid-19 crisis corresponded to an accelerated transformation of work organisation, with a very significant development of teleworking, which had a diversity of consequences [10]. Finally, for professions considered essential, such as healthcare workers, the Covid-19 crisis has been characterised by a continuation of face-to-face activity, most often under deteriorated working conditions [44,45]. In short, the way in which the health crisis was experienced depended to a large extent on one’s occupation.

One possibility is that these different experiences influence attitudes and behaviour towards vaccination and the various actors involved in the answer to the epidemic. With regard not to vaccination but to the acceptance of lockdowns, studies in France have shown very significant differences in support depending on whether individuals worked outside the home, teleworked or were off work because of a lockdown [28]. Our results show that *employees* and *manual workers* are less confident about vaccination and less willing to vaccinate than *managers and higher intellectual occupations*. Yet, statistically, these are also the categories whose experience of the pandemic were materially worse. We can therefore suggest that working conditions may have an influence on confidence: when they are poor, they reduce job satisfaction and are likely to weaken people’s confidence, which can have an impact beyond the strict sphere of work [19,46]. This could explain why, in our results, employees who work in the public sector, and therefore benefit from a more protective status, are less likely to be against vaccination and more inclined to vaccinate.

One’s occupation is also likely to have a direct influence on people’s relationship with medical expertise and public health recommendations. This is well illustrated in studies focusing on healthcare workers. The available data show that they are more receptive to public health messages than the rest of the population taken as a whole [13]. This phenomenon can be explained in part by their initial training, but also by their daily acculturation to health issues. Their position as transmitters, disseminators and relays of “good practice” also contributes to their appropriation of medical expertise and public health messages, both in their work and in their private lives. However, healthcare professionals are not a homogenous group. Studies show that, in France as in other countries, there is a “professional gradient” (i.e., the continuous and hierarchical distribution of professional positions, based on criteria such as skill level, symbolic prestige, autonomy, or associated economic resources) in terms of adherence to vaccination, based on a pattern of doctors – nurses – nursing assistants [13] (Anonymous 1). Looking at all socio-professional groups, we found a comparable social gradient, where *managers and higher intellectual occupations* are more inclined to be in favour of vaccines than *intermediate professions*, which are themselves more inclined than *employees*. The distinctions observed in the field of healthcare, where the social division of labour places doctors in a dominant position and relegates nursing assistants (and, to a lesser extent, nurses) to the “dirty work” [47,48] is likely to be observed in other professions as well. The feeling of being devalued at work, captured here by the position on the social ladder of occupations, could thus explain the lesser adherence to what may appear to be a strong social injunction imposed by people higher up the organisational ladder [5,15,49]. It should be noted that these phenomena of devaluation at work were particularly salient at the time of the Covid-19 crisis in France, which largely contributed to activating or reinforcing them. This can be seen, for example, in the analysis of responses among the various “essential workers”. There are major differences between nursing assistants and paramedics on the one hand, and pharmacists on the other. The former, having borne the full brunt of the massive influx of patients into hospitals, in a context of saturation of services and after several years of deterioration in their working conditions [50]. Various studies and media reports underline their helplessness and their feeling of being abandoned, a feeling largely symbolised by major material difficulties, such as the absence of masks or gowns [for a review of data see 23]. These trends are not specific to France [12,13]. Compared with pharmacists, who are more protected and have benefited from the period – including financially -, people in these occupations are much less likely to say they are in favour of vaccinations in general, to want to be vaccinated against Covid-19, to have confidence in the government and even in scientists. Similar differences can also be observed between doctors and nurses. It should be noted, however, that between these two categories (and for the healthcare professions in general) the differences in confidence in the government are much greater than those in confidence in scientists. This suggests that we need to go beyond the question of socialisation to health and look at work from the angle of its interplay with socialisation to State institutions and to politics.

### Work and institutional trust

The links between vaccination and the various dimensions of politics are now well documented in France as in many other countries [51,52]. In the case of a health crisis such as Covid-19, the interweaving of politics and health is particularly pronounced and salient: it is the government that announces the vaccination schedule, restrictions on movement or freedom, and so on. Differences in terms of partisan composition of these socio-professional groups could therefore explain some of our results. Unfortunately, the EpiCov study did not include political variables in its first waves. Future studies should investigate further how professional and political socializations interplay to bear on attitudes to health and medicine, as some have started to do [53]. However, it would be a mistake to limit our understanding of relationship to politics to partisanship. While partisanship has been found to be a significant determinant of attitudes to vaccines in many countries including France [23,51,52,54]studies also tend to show that trust in public institutions and decision-makers in general is an even more important determinant, especially in France [52].

Relationships to the State and public institutions vary greatly depending on one’s social class, of which occupation is an important part. Generally speaking, the working classes show a greater distrust of the State, which is reflected, for example, in a greater distrust of taxation than other classes [55]. For the French sociologist Alexis Spire, resistance to tax is partly explained by the “social production of ignorance”, which leads many taxpayers to be unaware of the existence of social assistance schemes. Other studies emphasise the specific relationship that the working classes have with state institutions. Compared to the middle and upper classes, they are more exposed to surveillance and repression by institutions (unemployment, prisons, etc.), more economically dependent on social benefit organisations, more dependent on public services and, at the same time, have a greater cultural distance from institutions, due to a lower level of cultural capital [56]. Our analysis also shows that members of the working classes are the most reluctant to vaccinate but also the least confident in the government. It is reasonable to suggest that, in addition to the specificity of the Covid-19 period during which the LREM-led government mainly benefited from support from the most affluent members of the public, their distance to the State participated in producing this mistrust. This hypothesis is based on the fact that there are differences between socio-professional groups at comparable levels in the social hierarchy. For example, *public sector clerical and service workers* are more favourable to vaccination in general than *corporate administrative workers* or *retail workers*. This relationship is also found among *managers and higher intellectual occupations* and among *intermediate professions*. Proximity to the State, including by being one of its employees, seems to limit distrust of the public health policies it promotes. This is in line with studies attesting to the specificity of individuals working for a public employer, who are more inclined to civic participation, altruism and social trust [57].

However, this relationship does not seem to hold true for confidence in the government. In fact, among *managers and higher intellectual occupations* and *intermediate professions*, those in the public service have less confidence in the government to limit the spread of the virus than those in the private sector. This observation invites us to distinguish between the State as an institution to which individuals remain attached, and the government, which tends to be seen as partisan and to be widely distrusted [58]. Perceptions of these public policies are certainly partly dependent on how the State or the government is viewed, but they are also dependent on the level of trust in other institutions (science, the police, schools, etc.) [59].

Trust in the various actors involved in vaccination policies is one of the main determinants of vaccine attitudes and behaviours [60,61]. In the construction of trust, people’s occupation can play an important role. Although political issues are less directly discussed there than with family or friends, exchanges are frequent – especially when it comes to salient issues [62] – and can contribute to the construction of shared representations. It is also a place where collective dynamics can be created, whether political [63,64] or directly related to health, as can be seen in the mobilising effects of blood donation drives in the workplace [65]. In the case of Covid-19, the link between vaccination and work is particularly obvious insofar as vaccination, or at least the provision of a negative covid test, has been made compulsory to access the workplace in many countries. As a consequence, for a large number of people, the question of vaccination was directly linked to the ability to continue working and to the State’s ability to interfer with their working practices.

In sum, a more detailed analysis of occupational groups reveals notable differences in workers’ attitudes toward political institutions. This confirms the central role of work in shaping political attitudes and behaviors, and extends the analysis beyond voting—an area that has attracted considerable scholarly attention in recent years [66–69].

The data at our disposal do not allow us to measure precisely the mechanism linking these socio-professional categories and our outcomes, nor to adopt a truly multidimensional approach to work that would allow a full assessment of its impact on political attitudes. On the other hand, they do open up avenues for reflection, particularly on the effect of the employer. We have seen that people working in the public service tend to be more in favour of vaccination, while being less confident in the government. How can this be explained? One possibility is that public servants associate vaccines less with the government than other professions do. In this sense, there would be a greater decorrelation between the judgement on politicians and public health policies themselves – as was observed among individuals who were very opposed to the government but who, while strongly criticising it, were actively involved in ensuring that lockdowns were respected in France [70]. As they work (directly or indirectly) for public authorities, these people might be more likely to observe that the content of their work goes far beyond the simple implementation of government directives. This could lead to a finer understanding of the workings of political and administrative institutions, and a better grasp of the balance of power, checks and balances and the relative autonomy from political pressure that some actors within the State enjoy. To explain the greater distrust of the government among public servants, we can also put forward the hypothesis that they experience the consequences of government policies more directly in their work than those working in the private sector. This is crucial in a context of decrease in investment in public services. For example, between 2006 and 2021 in France, the real average pay of public servants has fallen by 0.9% since 2009, whereas it has risen by 13.1% in the private sector [71]. We can therefore assume that a teacher who sees his or her job deteriorating will have less confidence in the government to manage the epidemic.

### Limitations section

Despite the many strengths of this survey, several limitations should be acknowledged. First, sample attrition led to some individuals dropping out between waves. Second, it is important to note that the data are self-reported, and may be subject to recall or reconstruction biases. Third, the survey includes few variables related to politicization, which is a crucial factor for understanding attitudes toward vaccines and health, including in France [52]. Likewise, the analysis could be enriched by more detailed data on actual working conditions, which also play a significant role in shaping vaccine-related attitudes and behaviors [12]. Finally, it would have been interesting to know whether individuals regretted having been vaccinated, particularly in light of the introduction of the health pass [32,53,54,72,73].

## Supporting information

S1 File Tables A1–A2: Composition of samples, wave 2 and wave 3, % column. (XLSX)

S2 File Tables B1–B2: Cross-tabulations wave 2 and wave 3. (XLSX)

S3 File Table 2bis: Focus on key occupations (Wave 3). (XLSX)

S4 File Tables C1–C4: binomial logit regressions wave 2 (multiple Ref.). (XLSX)

S5 File Tables D1–D4: binomial logit regressions wave 3 (multiple Ref.). (XLSX)

S6 File Tables E1–E4: ordinal logistics regressions wave 2 (multiple Ref.). (XLSX)

S7 File Supplementary tables F1–F4: ordinal logistics regressions wave 3 (multiple Ref.). (XLSX)
